# Supplementary material for: Aspergillus fumigatus Can Display Persistence to the Fungicidal Drug Voriconazole
Source: Microbiol Spectr. 2023 Mar 13;11(2):e04770-22. doi: 10.1128/spectrum.04770-22 (PMC10100717; doi:10.1128/spectrum.04770-22)
Supplement: Supplemental file 1 — Supplemental material. Download spectrum.04770-22-s0001.pdf, PDF file, 2.2 MB [file spectrum.04770-22-s0001.pdf]

**SUPPLEMENTARY FIGURE LEGENDS**

**Fig. S1.**
Disc diffusion screening of the PD-47 collection of isolates (Table S1 at https://doi.org/10.5281/zenodo.7533742) on RPMI plate with 10 µL of 0.8 mg/mL voriconazole. Only 5 isolates could grow colonies in the inhibition halos. Upon re-inoculation of the colonies of the halo (grown in the absence or presence of voriconazole, as detailed), PD-254, PD-256 and PD-266 covered the entire plate. As explained in the text those strains were classified as resistant or potentially heteroresistant. In contrast, PD-9 and PD-104 CoHs showed upon re-inoculation the same level of growth as the original isolates. These strains were classified as persisters.

**Fig. S2.**
**A)** The standardized E-test strip provided the same results as disc diffusion assays: the persister isolates PD-9 and PD-104 developed colonies in the halo of inhibition, and the non-persister isolates ATCC and PD-60 did not. E-test was performed according to Biomerieux instructions and repeated three independent times. **B)** Two CoHs from PD-9 and PD-104 were picked and sequentially passaged on PDA without drug. Every second passage the spores were inoculated in a new disc diffusion assay to check the level of resistance (diameter of the halo) and persistence (appearance of colonies in the halo). After 7 passages both CoHs from both isolates showed the same level of persistence, demonstrating that this phenotype is stable.

**Fig. S3.**
Disc diffusion screening of the PD-47 collection of isolates. Three days after inoculation, the disc containing voriconazole (10 µL of 0.8 mg/mL) was substituted for another one containing *Aspergillus* minimal medium. A few colonies were able to grow colonies in the halos after the switch, suggesting that a few conidia remain viable. Only three strains PD-9 and PD-104 and PD-259 grew colonies before the switch. Of those, only PD-9 and PD-104 grew additional colonies after the switch.

**Fig. S4.**
Independent repetitions of the experiment shown in Figure 1B. The wells of 1X, 2X and 3X MIC of voriconazole for the isolates ATCC, PD-9, PD-60 and PD-104 were imaged under the microscope. The non-persister isolates ATCC and PD-60 only displayed limited microscopic growth at 1X MIC. In contrast, the persister isolates PD-9 and PD-104 displayed microscopic growth at 1X, 2X and 3X supra-MIC concentrations. Scale bar= 132.5 µm.

**Fig. S5**
**A)** Photos taken at the site of ATCC conidia inoculation on an RPMI agar plate showed that germlings are visible after 8h of incubation at 37 °C, and short hyphae have developed after 16 hours of incubation. Agar was cut out an inverted on a cover slip containing a drop of PBS. Images were taken on a Leica SP8 inverted microscope. Images were processed using Fiji [129]. **B)** There was no difference in the germination rate or ratio of the isolates ATCC, PD-9, PD-60 and PD-104. **C)** Measurement of growth rate of persister and non-persister isolates on solid RPMI showed that the strains have no difference in basal growth. **D)** Measurement of growth rate of persister and non-persister isolates on liquid RPMI showed that the strains have no difference in basal growth. **E)** Quantification of CoHs formed on rich media under hypoxia.

**Fig. S6.**
Disc diffusion screening of the PD-47 collection of isolates (Table S1 at https://doi.org/10.5281/zenodo.7533742) on RPMI plate with 10 µL of 3.2 mg/mL itraconazole. Isolates RC (as expected) and PD-256 did not form an inhibition halo, reflecting that they are resistant to itraconazole. Isolates PD-104 and PD-266 could grow colonies in the halo. PD-104 formed upon re-inoculation a halo of the same size and similar number of CoHs as the original isolate, suggesting that it is persistence to itraconazole. The isolate PD-266 nearly covered the inhibition halo upon reinoculation, suggesting an increment in its MIC and therefore that it is potentially heteroresistant to itraconazole, as previously shown for voriconazole.

**Fig. S7.**

**A)** MICs of itraconazole and isavuconazole for the isolates ATCC, PD-9, PD-60 and PD-104, as calculated by broth dilution assay. **B)** The isolates PD-9 and PD-104 displayed some microscopic growth at 2X the MIC concentration for both azoles, whilst the isolates ATCC and PD-60 did not grow at all and only resting conidia could be found. Scale bar= 132.5 µm. **C)** The entire content of the wells containing the highest concentration (8 µg/mL) if each azole were inoculated on PDA plates and incubated for 48 h at 37 °C. A noticeable ratio of viable conidia were detected for the isolates PD-9 and PD-104. Each experiment was repeated twice independently. The graph represents the means and SD.

**Fig. S8.**

**A)** A nylon membrane was placed on the RPMI plate. Conidia were inoculated on the membrane and the disc containing voriconazole (10 µL of 0.8 mg/mL) was also put on it. As in a normal disc diffusion assay, an inhibition halo was formed, inside of which the persister strain PD-104 was able to grow small colonies, but the non-persister strain A1160 was not. **B)** RT-PCR of six selected DEGs detected in the transcriptomic analysis confirmed their differential expression, validating the RNA-seq. Additionally, the expression of the *adg3* gene was confirmed to be elevated in persister growth. The graphs display the mean and SD FC of 2 biological replicates (with three technical replicates). No Drug means sample obtained from a plate without voriconazole disc, Low Drug means sample collected at mid-distance of the inhibition halo and Persister means samples taken from colonies inside the inhibition halo (as shown in Fig. 5).

**Fig. S9.**

**A)** A network of interacting proteins was detected by STRING ($p$=1.84e-08) when using as query the 64 genes upregulated only in persistence in PD-104. Seventeen proteins related with metabolism formed a node, formed a node suggesting a specific metabolic response during persister growth. The predicted function of these genes is shown in the figure. **B)** Within the 18 genes that were upregulated in all comparisons, a tight node of 6 interacting proteins, related with ergosterol production, was detected by STRING ($p$<1.0e-16). The predicted function of these genes is shown in Table 2.

**Fig. S10.**

**A)** Representative disc diffusion plates carried out with the collection of isolates from the TAU medical centre. Six isolates were able to form CoHs, which did upon reinoculation form halos of the same size (MIC did not increase) and create a similar number of CoHs. **B)** Survival curves of larvae infected with $10^4$ or $5\times10^4$ conidia of the isolates ATCC, PD-60 (non-persisters), PD-9 or PD-104 (persisters). All strains killed larvae at a similar rate, demonstrating that they are equally virulent. For $10^4$, three independent experiments were done with 15-20 larvae/isolate in each. For $5\times10^4$, two independent experiments were done with 10-15 larvae/isolate in each. **C)** Resazurin was added to the broth dilution RPMI plate at a 0.002% (w/v) final concentration. The plate was incubated for 24 hours in a Tecan Infinity M-Plex plate reader and fluorescence was measured every 30 minutes with an excitation wavelength of 544 nm and reading emission at 590 nm using the i-control 2.0 software. The read for each well at each time point was normalize as follows: (read well time X– read 8 µg/mL voriconazole time X)/read well at time 0. The time point with the best dynamic range of values in the 1X, 2X and 3X MIC was found to be 20 hours, which was selected to determine the metabolic activity. A value of 0.1 was assigned as background and values above 1 were detected in sub-MIC (macroscopic growth). At 1X MIC, a slight metabolic activity could be detected for both the non-persister ATCC and the persister isolates PD-9 and PD-104. At 2X and 3X the MIC, slight metabolic activity could only be detected for the persister isolates. The experiment was performed once with two biological replicates, the graph represent the means and SEM.

Fig. S1

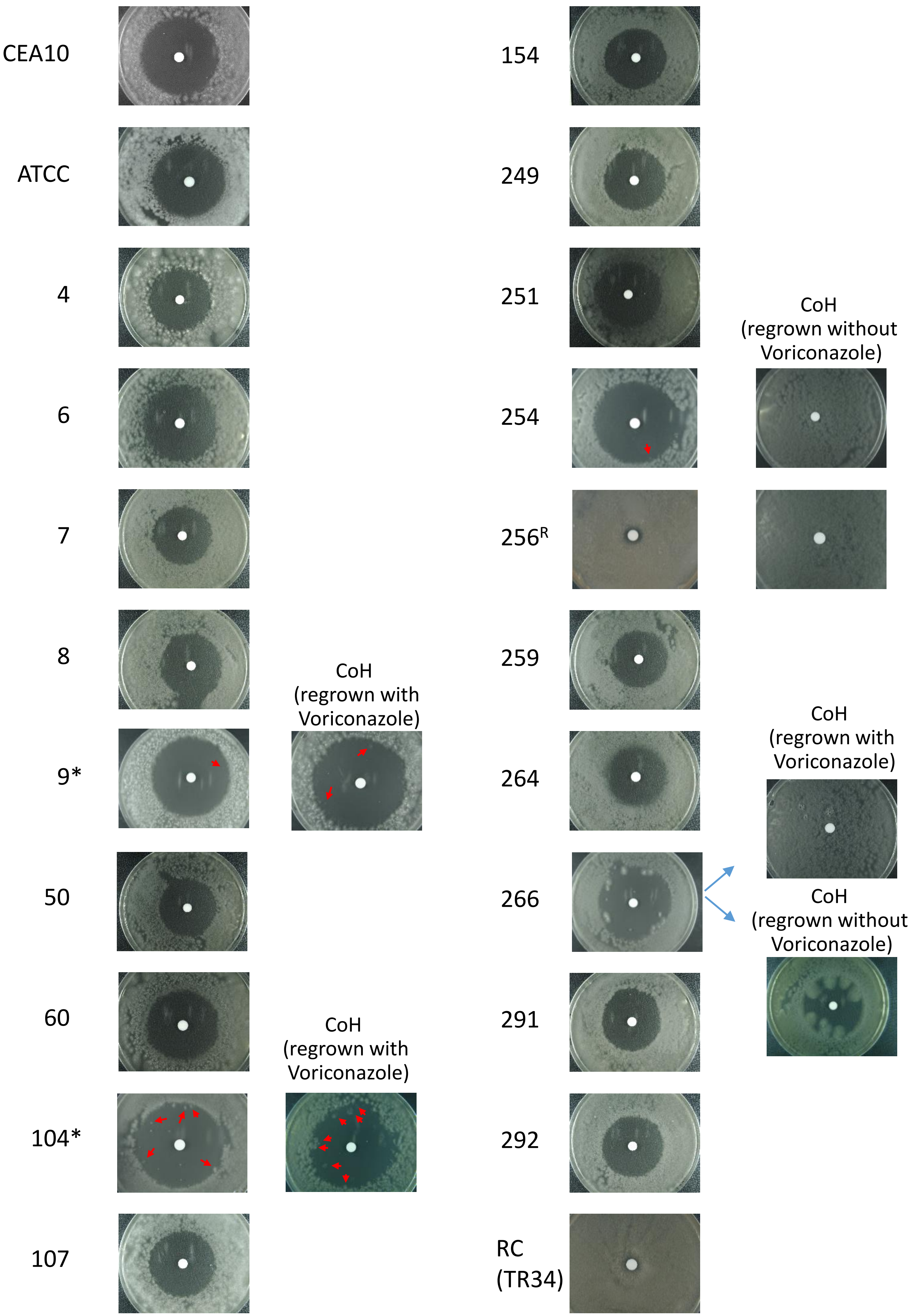

CEA10
ATCC
4
6
7
8
9*
CoH (regrown with Voriconazole)
50
60
104*
CoH (regrown with Voriconazole)
107
154
249
251
CoH (regrown without Voriconazole)
254
256R
259
264
CoH (regrown with Voriconazole)
266
CoH (regrown without Voriconazole)
291
292
RC (TR34)

Fig. S2

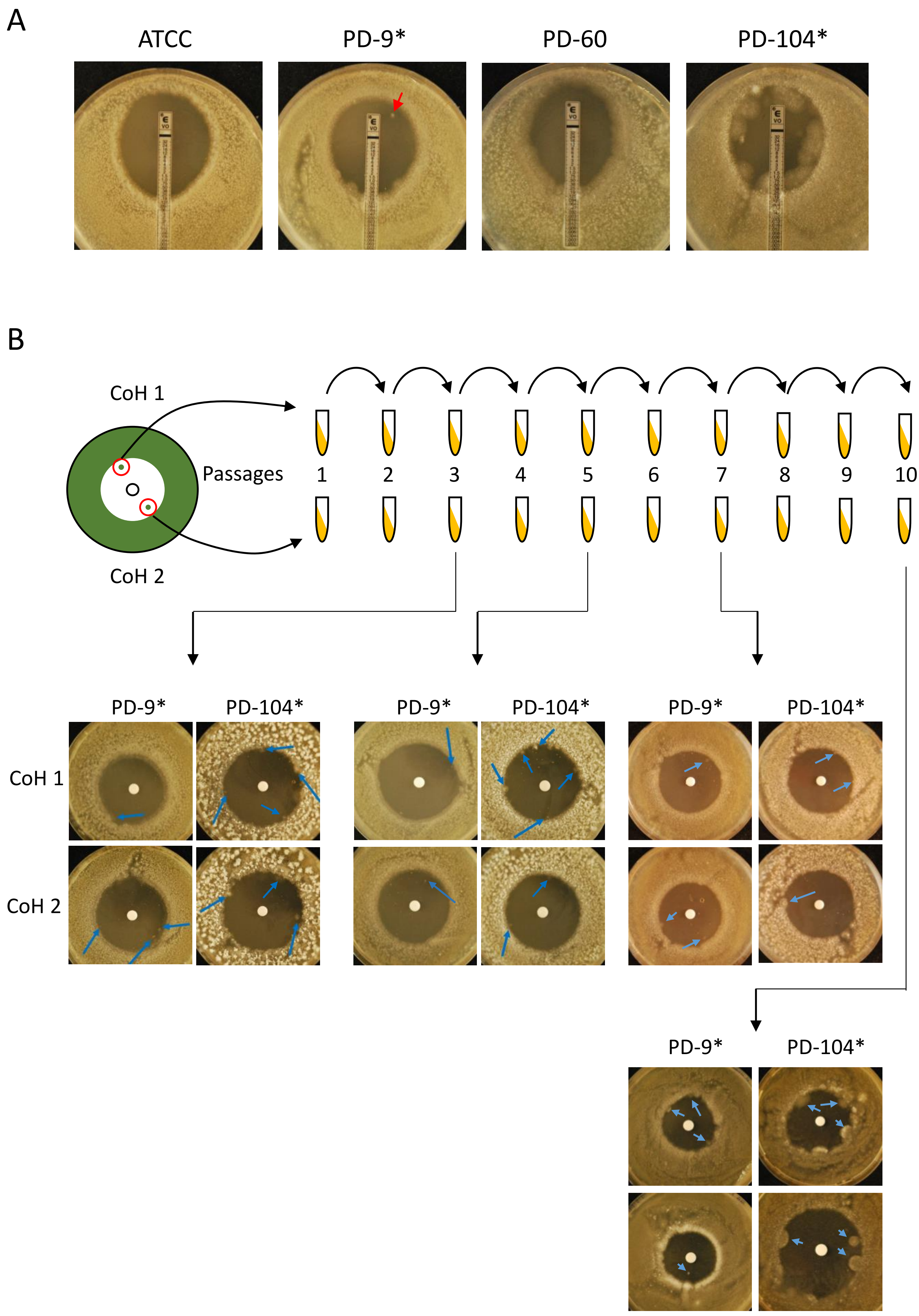
A
ATCC
PD-9*
PD-60
PD-104*
B
CoH 1
Passages
CoH 2
1
2
3
4
5
6
7
8
9
10
PD-9*
PD-104*
CoH 1
CoH 2
PD-9*
PD-104*
PD-9*
PD-104*
PD-9*
PD-104*

Fig. S3

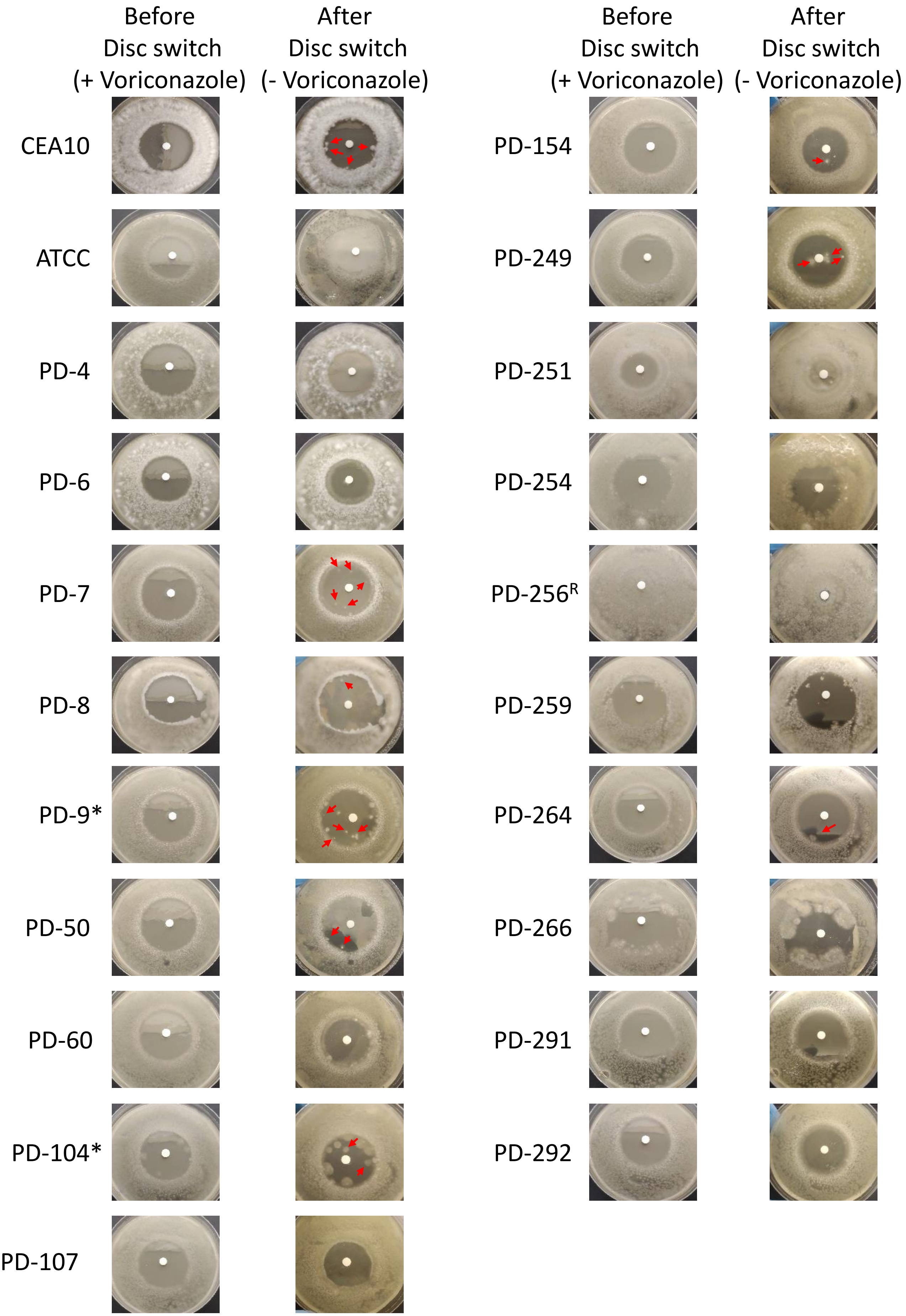
Before
Disc switch
(+ Voriconazole)
After
Disc switch
(- Voriconazole)
Before
Disc switch
(+ Voriconazole)
After
Disc switch
(- Voriconazole)
CEA10
ATCC
PD-4
PD-6
PD-7
PD-8
PD-9*
PD-50
PD-60
PD-104*
PD-107
PD-154
PD-249
PD-251
PD-254
PD-256R
PD-259
PD-264
PD-266
PD-291
PD-292

Fig. S4

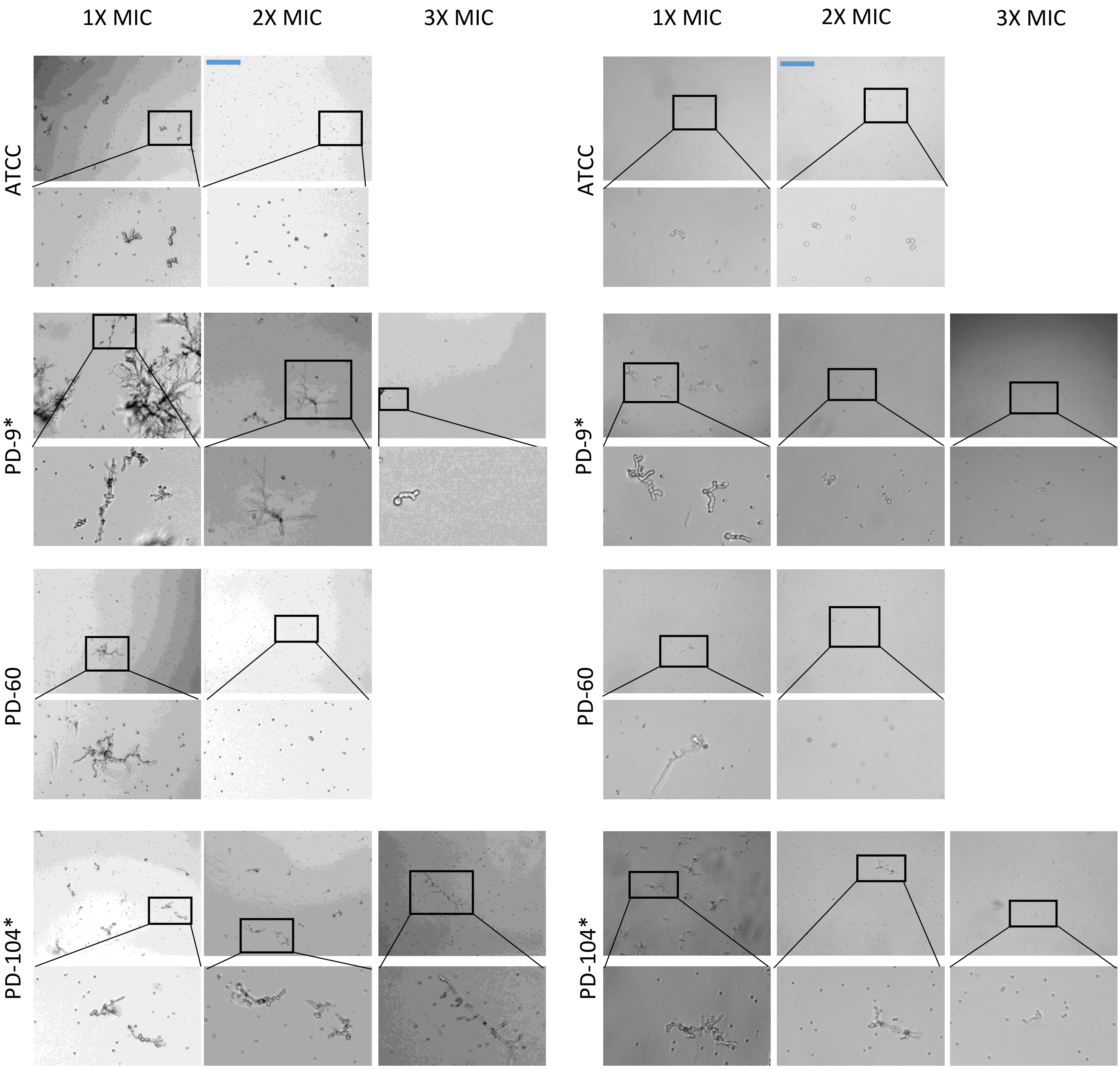
1X MIC
2X MIC
3X MIC
1X MIC
2X MIC
3X MIC
ATCC
PD-9*
PD-60
PD-104*
ATCC
PD-9*
PD-60
PD-104*

Fig. S5

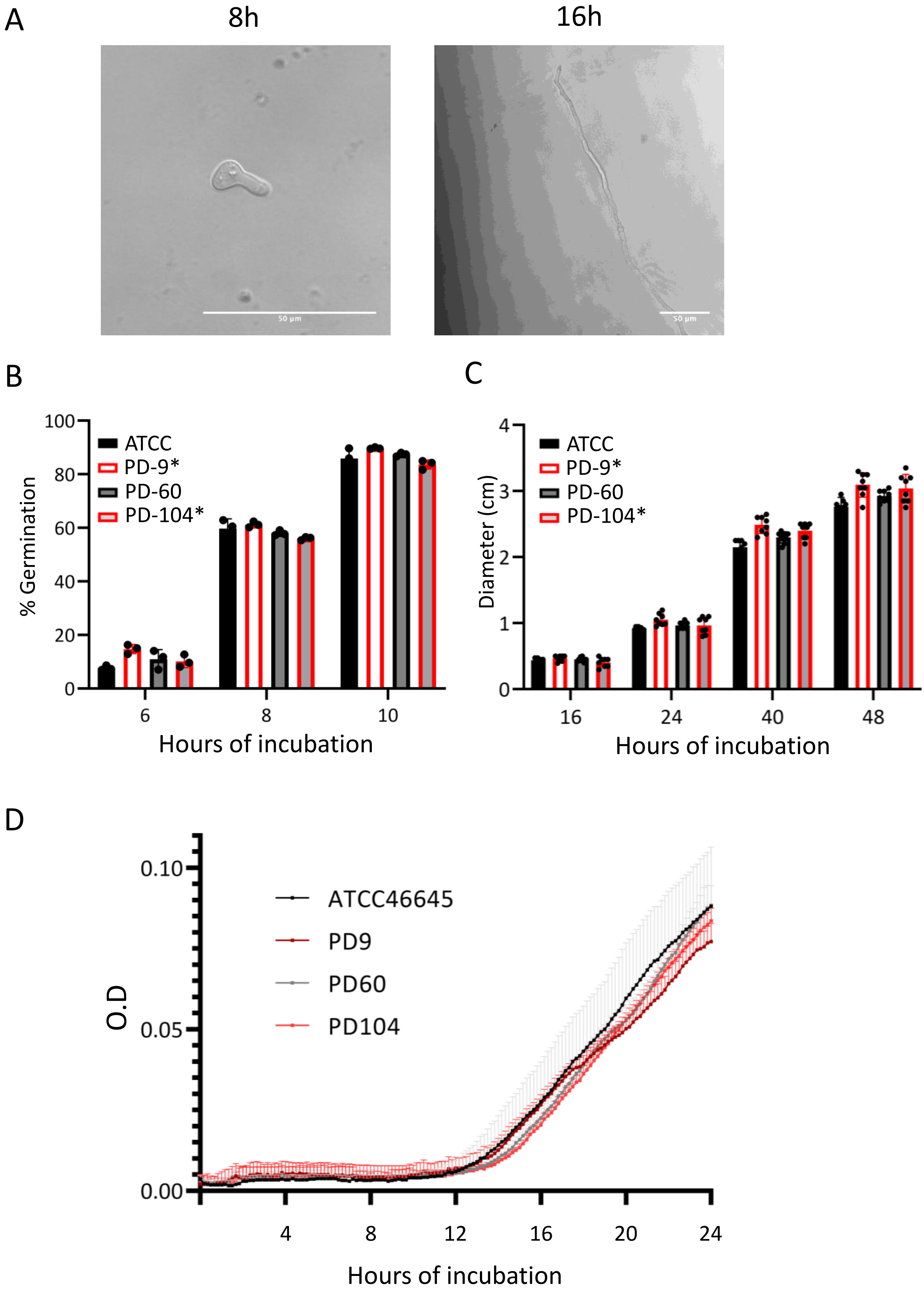

A
8h
16h
50 µm
50 µm
B
% Germination
100
80
60
40
20
0
ATCC
PD-9*
PD-60
PD-104*
6
8
10
Hours of incubation
C
Diameter (cm)
4
3
2
1
0
ATCC
PD-9*
PD-60
PD-104*
16
24
40
48
Hours of incubation
D
O.D
0.10
0.05
0.00
ATCC46645
PD9
PD60
PD104
4
8
12
16
20
24
Hours of incubation

Fig. S6

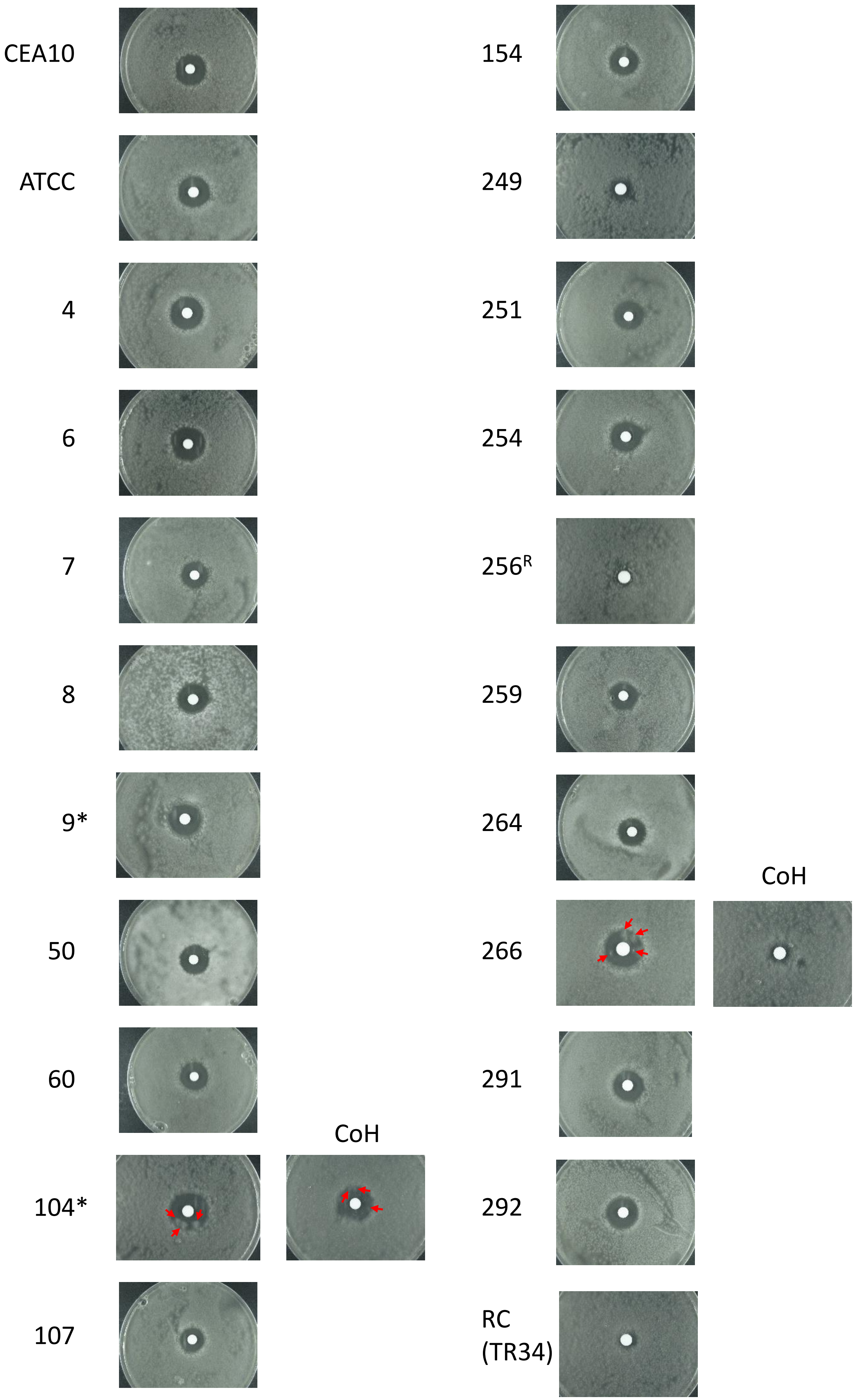

CEA10
ATCC
4
6
7
8
9*
50
60
104*
CoH
107
154
249
251
254
256R
259
264
266
CoH
291
292
RC
(TR34)

Fig. S7

A

| MIC | Itraconazole (µg/mL) | Isavuconazole (µg/mL) |
|---|---|---|
| ATCC | 0.5 | 1 |
| PD9* | 0.5 | 1 |
| PD60 | 0.5 | 1 |
| PD104 * | 0.5 | 1 |

B

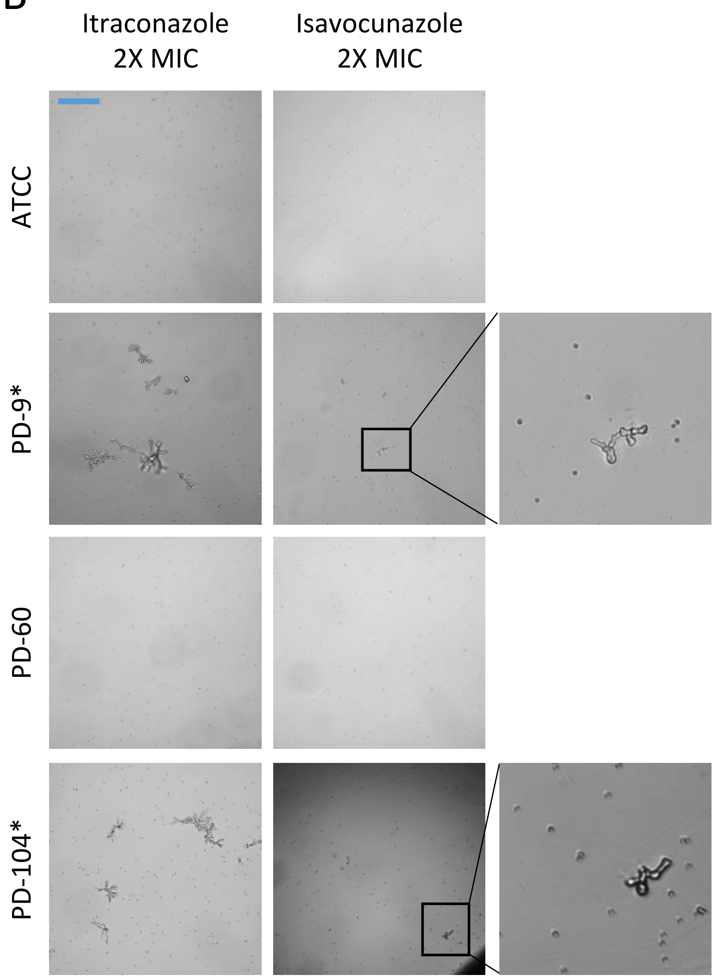


C

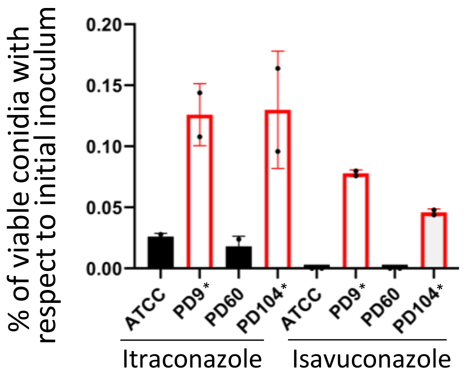

Fig. S8

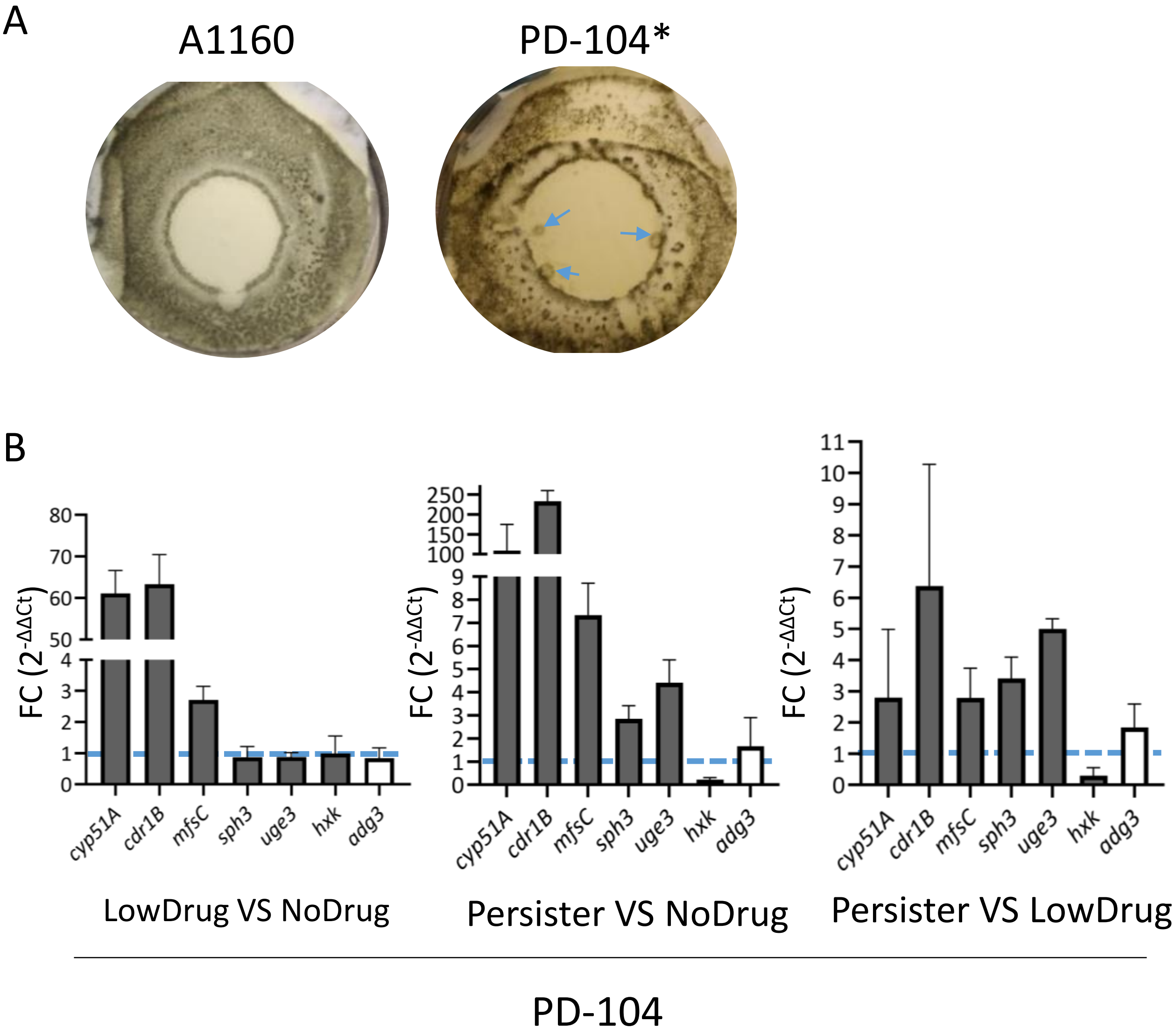
A
A1160
PD-104*
B
FC (2^-ΔΔCt)
cyp51A
cdr1B
mfsC
sph3
uge3
hxk
adg3
LowDrug VS NoDrug
Persister VS NoDrug
Persister VS LowDrug
PD-104

# Fig. S9

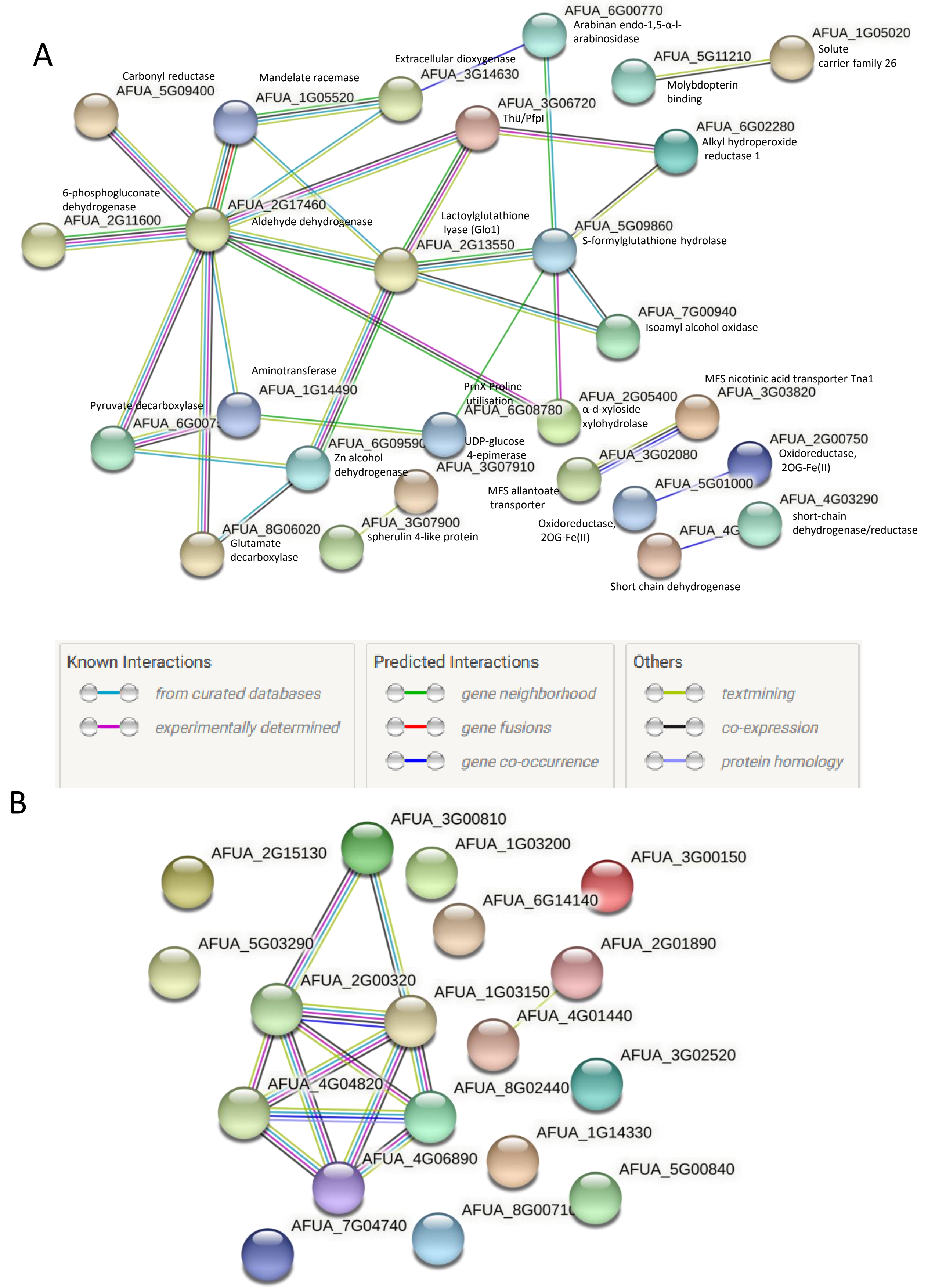
A
AFUA_6G00770
Arabinan endo-1,5-α-l-arabinosidase
AFUA_1G05020
Solute carrier family 26
AFUA_5G11210
Molybdopterin binding
Extracellular dioxygenase
AFUA_3G14630
Carbonyl reductase
AFUA_5G09400
Mandelate racemase
AFUA_1G05520
AFUA_3G06720
ThiJ/PfpI
AFUA_6G02280
Alkyl hydroperoxide reductase 1
6-phosphogluconate dehydrogenase
AFUA_2G11600
AFUA_2G17460
Aldehyde dehydrogenase
Lactoylglutathione lyase (Glo1)
AFUA_2G13550
AFUA_5G09860
S-formylglutathione hydrolase
AFUA_7G00940
Isoamyl alcohol oxidase
Aminotransferase
AFUA_1G14490
PrnX Proline utilisation
AFUA_6G08780
AFUA_2G05400
α-d-xyloside xylohydrolase
MFS nicotinic acid transporter Tna1
AFUA_3G03820
Pyruvate decarboxylase
AFUA_6G007
AFUA_6G0959
Zn alcohol dehydrogenase
UDP-glucose 4-epimerase
AFUA_3G07910
AFUA_3G02080
MFS allantoate transporter
AFUA_2G00750
Oxidoreductase, 2OG-Fe(II)
AFUA_5G01000
Oxidoreductase, 2OG-Fe(II)
AFUA_4G03290
short-chain dehydrogenase/reductase
AFUA_4G
AFUA_8G06020
Glutamate decarboxylase
AFUA_3G07900
spherulin 4-like protein
Short chain dehydrogenase
Known Interactions
from curated databases
experimentally determined
Predicted Interactions
gene neighborhood
gene fusions
gene co-occurrence
Others
textmining
co-expression
protein homology
B
AFUA_3G00810
AFUA_2G15130
AFUA_1G03200
AFUA_3G00150
AFUA_6G14140
AFUA_5G03290
AFUA_2G01890
AFUA_2G00320
AFUA_1G03150
AFUA_4G01440
AFUA_3G02520
AFUA_4G04820
AFUA_8G02440
AFUA_1G14330
AFUA_4G06890
AFUA_5G00840
AFUA_8G0071
AFUA_7G04740

Fig. S10

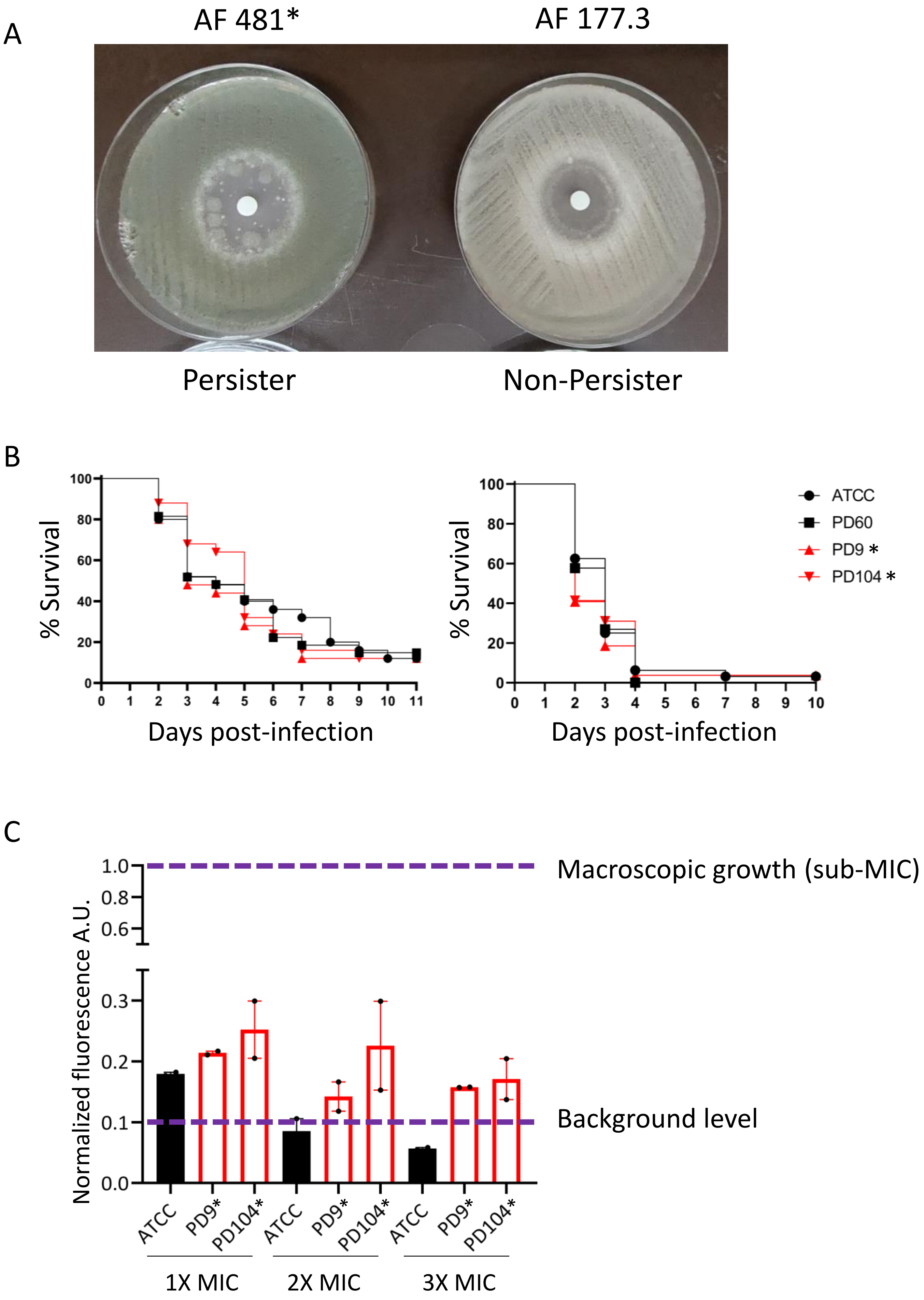
A
AF 481*
AF 177.3
Persister
Non-Persister
B
% Survival
Days post-infection
ATCC
PD60
PD9 *
PD104 *
C
Normalized fluorescence A.U.
Macroscopic growth (sub-MIC)
Background level
ATCC
PD9*
PD104*
1X MIC
2X MIC
3X MIC
